# Approximate Counting of Graphical Realizations

**DOI:** 10.1371/journal.pone.0131300

**Published:** 2015-07-10

**Authors:** Péter L. Erdős, Sándor Z. Kiss, István Miklós, Lajos Soukup

**Affiliations:** 1 Alfréd Rényi Institute of Mathematics, Hungarian Academy of Sciences, Budapest, Hungary; 2 Institute for Computer Science and Control, Hungarian Academy of Sciences, Budapest, Hungary; 3 Department of Algebra, University of Technology and Economics, Budapest, Hungary; Max F. Perutz Laboratories, AUSTRIA

## Abstract

In 1999 Kannan, Tetali and Vempala proposed a MCMC method to uniformly sample all possible realizations of a given graphical degree sequence and conjectured its rapidly mixing nature. Recently their conjecture was proved affirmative for regular graphs (by Cooper, Dyer and Greenhill, 2007), for regular directed graphs (by Greenhill, 2011) and for half-regular bipartite graphs (by Miklós, Erdős and Soukup, 2013).

Several heuristics on counting the number of possible realizations exist (via sampling processes), and while they work well in practice, so far no approximation guarantees exist for such an approach. This paper is the first to develop a method for counting realizations with provable approximation guarantee. In fact, we solve a slightly more general problem; besides the graphical degree sequence a small set of forbidden edges is also given. We show that for the general problem (which contains the Greenhill problem and the Miklós, Erdős and Soukup problem as special cases) the derived MCMC process is rapidly mixing. Further, we show that this new problem is *self-reducible* therefore it provides a *fully polynomial randomized approximation scheme* (a.k.a. FPRAS) for counting of all realizations.

## Introduction

In the Age of the Internet, network theory has been undergoing exponential growth. One of its important problems is to algorithmically construct networks (or *graphs*) with predefined parameters, or to uniformly sample networks with these parameters. For general background, the interested reader can turn to the now-classic book of Newman, Barabási and Watts ([1]) or to the more recent book of Newman ([2]).

One of the earliest and still most important problems in graph theory is uniformly sampling all possible graph realizations of given degree sequence. (For the definitions see Section “Degree sequences”.) One possible method for this is a simple MCMC approach (proposed by Kannan, Tetali and Vempala [3]); take an arbitrary realization of the degree sequence, then perform a series of randomly chosen local transformations (called *swap* or *switch*). They conjectured that the process is *rapidly mixing*, i.e., a random realization is achieved after polynomial many steps.

The first result with a flawless proof in connection with this conjecture is due to Cooper, Dyer and Greenhill (2007, [4]) for the special case when the degree sequence is regular. Greenhill proved in 2011 the analogous result for (in- and out-)regular directed graphs ([5]). In 2013 Miklós, Erdős and Soukup proved the conjecture for *half-regular* bipartite graphs ([6]). Here the degree sequence on one class is regular, while there is no constraint on the other class. (A comprehensive survey on the topic is [5] or is [6].)

In modern network applications sampling the solutions is just one requirement. Sometimes the actual number of all solutions (or at least a good approximation of it) is also important. It is well known (Jerrum, Valiant and Vazirani 1986, [7]) that for *self-reducible counting problems* a rapidly mixing sampling method also provides a quick estimation of that number (with small relative error and with very high probability). Unfortunately none of the sampling problems listed above belongs to this class.

The main purpose of this paper is to remedy this imperfection. For that end we introduce a slightly more general degree sequence problem, which has all the good characteristics of the sampling procedures above (including their rapidly mixing nature), furthermore, which belongs to the class of self-reducible counting problems. This new problem is a common generalization of the regular directed graph and of the half-regular bipartite graph cases. Therefore, showing the rapidly mixing nature of the corresponding MCMC procedure provides new proofs for both problems. We prove only the existence of a polynomial upper bound on the mixing time, but do not prove the tight upper bound in Greenhill’s theorem in [5].

In Section “Degree sequences” we recall the known definitions and facts on degree sequences problems in simple graphs. Then we introduce and study in full generality our proposed new *restricted degree sequence* (or **ReDeSe** for short) problem, where we deal with forbidden edges. Next, we study a specific instance of the general ReDeSe problem: bipartite degree sequences with a forbidden (but not necessarily perfect) 1-factor and a forbidden (but maybe empty) star.

In Section “Sampling” we first discuss some known results to sample degree sequence realizations. Then, we formulate our main result (Theorem 10): the proposed MCMC process on half-regular bipartite degree sequences with a well-defined small forbidden edge set is rapidly mixing. Our proof is based on Sinclair’s *multicommodity flow method* ([8]), and follows closely the proof in [6]. We discuss the similarity between the two proofs in this section, while in Sections “Milestones” and “The analysis” we study the details of our new Markov chain approach which require different treatment.

In Section “Counting” we show that the studied sampling problem leads to a self-reducible counting problem. Therefore, our almost uniform sampling method provides a good approximation on the size of the set of all realizations, strengthening also Greenhill’s result on regular directed graphs ([5]), and Miklós, Erdős and Soukup’s result on half-regular bipartite graphs ([6]).

## Degree Sequences

In this paper, every graph is assumed to be *simple*; there are no loops or multiple edges.

### Degree sequences and realizations

Let *V* be a labeled set of *n* elements. The *degree sequence* **d**(*G*) of a graph *G* = (*V*, *E*) is the sequence **d**(*G*)_*i*_ = *d*(*v*_*i*_) of its vertex degrees. A non-negative integer sequence **d** = (*d*_1_, …, *d*_*n*_) is *graphical* iff **d**(*G*) = **d** for some simple graph *G*, and then *G* is a *graphical realization* of **d**.

The first successful approach to decide whether a degree sequence is graphical is due to Havel ([9]), his simple but surprising observation provides immediately a greedy algorithm to build such a realization. His work was rediscovered independently later by Hakimi ([10]). Their method is based on the so-called *swap* operation. (The expressions *switch* or *rewiring* are also widely used. In this paper the word *switch* will be used for a similar, but slightly more general, operation.) The *swap* operation is defined as follows:

Let *G* be a simple graph and assume that *a*, *b*, *c* and *d* are different vertices. Furthermore, assume that (*a*, *c*), (*b*, *d*) ∈ *E*(*G*) while (*b*, *c*), (*a*, *d*) ∉ *E*(*G*). Then

$$E\left(G^{\prime }\right)=E\left(G\right)\setminus \left\{\left(a,c\right),\left(b,d\right)\right\}\cup \left\{\left(b,c\right),\left(a,d\right)\right\}$$ (1)

is another realization of the same degree sequence. We denote this operation by *ac*, *bd* ⇒ *bc*, *ad*. Havel’s nice observation is the following:

**Lemma 1** (Havel, [9]). *Assume that in graph G vertex v is adjacent to vertex x but not to vertex y. Assume furthermore that d*(*x*) ≤ *d*(*y*). *Then one can find a swap operation which produces a new graphical realization G′ of the degree sequence* **d**(*G*) *with the property:* Γ′(*v*) = Γ(*v*) ∖ {*x*} ∪ {*y*}. *(These are the corresponding neighborhoods of vertex v.)*

The analogous notions for bipartite graphs are the following: if *B* is a simple bipartite graph then its vertex classes will be denoted by *U*(*B*) = {*u*_1_, …, *u*_*k*_} and *W*(*B*) = {*w*_1_, …, *w*_ℓ_}, and we keep the notation *V*(*B*) = *U*(*B*) ∪ *W*(*B*). The *bipartite degree sequence* of *B*, **D**(*B*) is defined as follows:

$$\begin{array}{c} D\left(B\right)=((d\left(u_{1}\right),\ldots ,d\left(u_{k}\right)),(d\left(w_{1}\right),\ldots ,d\left(w_{\ell }\right))). \end{array}$$

We can define the swap operation for bipartite realizations similarly to Eq (1) but we must take some care: it is not enough to assume that (*b*, *c*), (*a*, *d*) ∉ *E*(*G*) but we have to know that *a* and *b* are in one vertex class, and *c* and *d* are in the other.

To make clear whether a vertex pair is not forbidden to be an edge we will call a vertex pair a **chord** if it can hold an actual edge in a realization. Those pairs that cannot accommodate an edge are **non-chords**. (For example, pairs from the same vertex class of a bipartite graph are non-chords.) It can also be found in [11, Theorem 6].

Denote $\vec{G}$ a directed graph (no parallel edges, no loops, but oppositely directed edges between two vertices are allowed) with vertex set $X(\vec{G})=\{x_{1},x_{2},\ldots ,x_{n}\}$ and edge set $E(\vec{G})$. For every vertex *v* we associate two numbers: the *in-degree* and the *out-degree* of *v*.

Instead of introducing the matching definitions, we will apply the following *representation* of the directed graph $\vec{G}:$ let $B(\vec{G})=(U,W;E)$ be a bipartite graph where each class consists of one copy of every vertex of $\vec{G}$. The edges adjacent to a vertex *u*_*x*_ in class *U* represent the out-edges from *x*, while the edges adjacent to a vertex *w*_*x*_ in class *W* represent the in-edges to *x* (so a directed edge *xy* corresponds the edge *u*_*x*_ *w*_*y*_). If a vertex has zero in- (respectively out-) degree in the directed version, then we delete the corresponding vertex from $B(\vec{G}).$ (Actually, this representation is an old trick used already by Gale [12].) There is no loop in our directed graph, therefore there is no (*u*_*x*_, *v*_*x*_) type edge in its bipartite realization—these vertex pairs are non-chords.

Consider two different realizations, *G* and *H*, of the same degree sequence (either simple or bipartite one). It is a well-known fact that the first can be transformed to the second one (and vice versa) with consecutive swap operations. Formally, there exists a series of realizations *G* = *G*_0_, …, *G*_*i*−1_, *G*_*i*_ = *H*, such that for each *j* = 0, …, *i*−1 there exists a swap operation which transforms *G*_*j*_ into *G*_*j*+1_.

For simple graphs this was proved already in 1891 by Petersen [13]. It can be shown that lemma 1 also provides a solution via the so-called *canonical realizations*. The analogous result for bipartite graphs (with possible multiple edges but no loops) is due to Ryser ([14]). For simple bipartite graphs this is common-knowledge.

For directed graphs an analogous result is known. It was discovered by Kleitman and Wang (see [15]) and later rediscovered in [16]. It is important however to recognize that in case of directed graphs the “classical” Havel-type swap operation is not always adequate. To see this, it is enough to consider an example with three vertices: each vertex incident to one in-edge and one out-edge. There are exactly two possible realizations of this directed degree sequence, therefore a swap operation with six chords is necessary: we exchange three edges with three non-edges in one step. In papers [15, 16] it was shown that this extra operation is always sufficient.

### Restricted degree sequences

In this paper we study the following common generalization of all previously mentioned degree sequence problems:

The **restricted degree sequence** (i.e. ReDeSe) problem **d** ^ℱ^ consists of a degree sequence **d** and a set ℱ⊂(V2) of **forbidden** edges. The problem is to decide whether there is a simple graph *G* on *V* with the given degree sequence and with *E*(*G*) ∩ ℱ = ∅.

It is clear that this problem is essentially identical with Tutte’s f-factor problem [17]: the f-factor to be found is our degree sequence while the graph what the f-factor is searched for is the complement of the forbidden edges. Therefore Tutte’s theorem and the famous blossom algorithm of Edmonds apply nicely for the ReDeSe problem. However the focus of our approach is quite different from the f-factor problem: at first we are interested several (or all) solutions of the ReDeSe problem instead of finding one solution, and often enough we want to find “typical” solutions. At second: this sampling problem seems to be hopeless in general. In our studies we restrict ourself for carefully chosen small instances.

The **bipartite restricted degree sequence** problem **D** ^ℱ^ consists of a bipartite degree sequence **D** on (*U*, *W*), and a set ℱ ⊂ [*U*, *W*] of **forbidden** edges. The problem is to decide whether there is a simple bipartite graph *G* on *V* with the given degree sequence and with *E*(*G*) ∩ ℱ = ∅.

Clearly, a bipartite restricted degree sequence problem **D** ^ℱ^ on (*U*, *W*) is the restricted degree sequence problem **d** ^ℱ^′ on *U* ∪ *W*, where ℱ′ = ℱ ∪ [*U*]^2^ ∪ [*W*]^2^.

Furthermore, we already studied one instance of the bipartite restricted degree sequence problem, namely the bipartite representation of directed degree sequences: here ℱ is one 1-factor, which corresponds to the forbidden loops.

It is important to add that while the fundamental result of Jerrum, Sinclair and Vigoda on sampling perfect matchings in graphs ([18]) provides a uniform sampling approach for the possible realizations, their method is not useful in practice. That is the reason that so much effort has been made on this topic. We return to this issue at the end of Section “Counting”.

In the remaining of this subsection we study the general ReDeSe problem. The next subsection will be devoted to a particular bipartite restricted degree sequence problem which will play a central role later in the paper.

**Definition 2.** Let **d** ^ℱ^ be a restricted degree sequence problem and let *G* be a realization of it. The sequence of vertices 𝓒 = (*x*_1_, *x*_2_, …, *x*_2*i*_) is a **chord-circuit** if:

(D1) all pairs *x*_1_ *x*_2_, *x*_2_ *x*_3_, …, *x*_2*i*−1_ *x*_2*i*_, *x*_2*i*_ *x*_1_ are chords;
(D2) each of these chords is different.

A chord-circuit is **elementary** if

(D3) no vertex occurs more than twice;
(D4) when two copies of the same vertex exist, then their distance along the circuit is odd.

**Definition 3.** The chord-circuit 𝓒 is said to **alternate** in *G*, if the chords along 𝓒 are in turn edges and non-edges in *G*. (For example *x*_2*j*−1_ *x*_2*j*_ are edges for 1 ≤ *j* ≤ *i*, while the other chords are non-edges in *G*.)

Deleting the actual edges along 𝓒 from *G* and adding the other chords as edges constructs a new graph *G*′ which is again a realization of **d** ^ℱ^. This is a **𝓒-swap** and this operation is known in general as a **circular *C*_2*i*_-swap**.

Finally, two *different* vertices *x*, *y* of the alternating chord-circuit 𝓒 form a **PV-pair** if the distance of the vertices along the circuit (the number of chords between them) is odd and greater than 1. If all PV-pairs are non-chords (so they belong to ℱ), then this circular 𝓒-swap is called a **ℱ-compatible swap** or **ℱ-swap** for short.

The ℱ-swap is one of the central notions of this paper. When *i* = 2 then the circular *C*_4_-swap coincides with the classical Havel type swap. When *i* = 3 then we get back the notion of the **triangular *C*_6_-swap**, which occurs in connection with directed degree sequences (see [19]).

We define the **weight** of the ℱ-compatible circular *C*_2*i*_-swap as *w*(*C*_2*i*_) = *i*−1. This definition sets the weight of the classical Havel type swaps to 1 and the weight of a *C*_6_-swap to 2, which agree with the definitions used in paper [19]. Furthermore it is well known (see for example again [19]) that (*i*−1) Havel type swaps are needed to alternate the edges along *C*_2*i*_ in case of simple graphs with no forbidden edges. As we will see next the same applies for any elementary circular *C*_2*i*_-swap:

**Lemma 4.** *Let G be a realization of* **d** ^ℱ^ *and let the elementary chord-circuit 𝓒 of length* 2*i be alternating. Then the circular 𝓒-swap operation can be carried out by a sequence of ℱ-swaps of total weight i*−1.

In other words there exists a sequence *G* = *G*_0_, *G*_1_, …, *G*_ℓ_ of realizations such that for each *j* = 0, …, ℓ−1 there exists an ℱ-compatible swap operation from *G*_*j*_ to *G*_*j*+1_. The difference between *G* and *G*_ℓ_ is exactly the alternating circuit 𝓒. Finally, the total weights of those ℱ-swap operations is *i*−1. We will say that this swap sequence does **process** the prescribed circular swap operation.

*Proof*. We apply mathematical induction for the length of the chord-circuit: when *i* = 2 then the statement is trivial. Assume now that this is true for all circuits of length at most 2*i*−2. Then take an alternating elementary chord-circuit 𝓒 of length 2*i* in a realization of **d** ^ℱ^.

If each PV-pair in 𝓒 is a non-chord, then the circular *C*_2*i*_-swap itself is a ℱ-swap of weight *i*−1. So we may assume that there is a PV-pair *uv* in 𝓒 which is a chord. This chord together with the two “half-circuits” of 𝓒 form chord-circuits 𝓒_1_ and 𝓒_2_ using the chords of the original circuit 𝓒 and twice the chord *uv*. One of them, say 𝓒_1_, is alternating. The length of 𝓒_1_ is 2*j* < 2*i* therefore there exists a ℱ-compatible swap sequence of total weight *j*−1 to process it. After the procedure the *status* of *uv* (the property of the chord whether it is an edge or a non-edge) will alter into the other status. With this new status of the chord the circuit 𝓒_2_ becomes an alternating one with length 2*i*+2−2*j*, so it can be processed with $\frac{2i+2-2j}{2}-1$ total weight—and after this procedure the chord *uv* is switched back to its original status. We found a swap sequence of total weight *i*−1 which finishes the proof.

**The space of all realizations of d** ^ℱ^: Consider now the set of all possible realizations of a restricted graphical degree sequence **d** ^ℱ^. Let *G* and *H* be two different realizations. The natural question, similar to the case of classical degree sequence problems, is whether *G* can be transformed into *H* using ℱ-swaps? The answer is affirmative:

**Theorem 5.** *The space* 𝔾 = (𝕍, 𝔼) *of all realizations of the restricted degree sequences problem* **d** ^ℱ^ *is connected*.

*Proof*. What we have to prove is the following: let *G* and *H* be two realizations of **d** ^ℱ^. Then we have to find a series of realizations *G* = *G*_0_, …, *G*_*i*−1_, *G*_*i*_ = *H*, such that for each *j* = 0, …, *i*−1 there exists an ℱ-swap from *G*_*j*_ to *G*_*j*+1_.

Consider the symmetric difference of the edge sets of the two realizations: Δ = *E*(*G*)△*E*(*H*). This set is two-colored by the original hosts of the edges: there are *G*-edges and *H*-edges. It is clear that for each vertex *v* in the graph 𝓖 = (*V*, Δ) the numbers of *G*-edges and *H*-edges incident to *v* are the same: *d*_*G*_(*v*) = *d*_*H*_(*v*). It is well known that this can be decomposed into alternating circuits 𝓒_1_, …, 𝓒_ℓ_.

We will use the notions of **circuit** and **cycle** in a simple graph *G* as usual: therefore a circuit is a chord-circuit where all chords are edges. A cycle is a circuit without repeated vertices. A circuit is **alternating** in Δ if the edges come in turns from *E*(*G*) and *E*(*H*). When this is the case then the corresponding chord-circuit in realization *G* (as well as in *H*) is also alternating.

We can find a decomposition, such that no circuit contains a vertex *v* twice and their distance *δ* (the number of edges between the copies is even. Indeed, if *δ* is even, then *δ* is at least four, consequently the vertex *v* splits the original circuit into two smaller, but still alternating circuits. Furthermore, if a circuit contains a vertex *v* at least three times, then there are at least two of them with even distance.

It is clear that any alternating circuit decomposition can be transformed into a decomposition where each (chord)-circuit is elementary with successive transformations. It is also clear that if, by chance, we start with a circuit decomposition of maximal number of circuits, then all circuits in this decomposition are automatically elementary. (Of course, finding such a decomposition may be very hard.)

The application of Lemma 4 proves that each circuit 𝓒 can be processed with |𝓒|/2−1 total weight. This finishes the proof.

It seems to be interesting that using a result from paper [19] one can determine the minimum weight of an ℱ-compatible swap sequence which transforms *G* into *H*, however we do not discuss this question here.

### Bipartite 1-Factor + 1 Star Restricted Degree Sequences

In the previous subsection we studied the restricted degree sequence problem in its full generality. However, our real interest lays in a quite simple case: **d** ^𝕱^ is called a **1-Factor + 1 Star Restricted Degree Sequence** problem (or **1F1S** problem for short), if

(Ψ) the set 𝕱 of forbidden edges is a bipartite graph where the edges are the union of an 1-factor and a star with center *s*.

Similarly, if **D** is a bipartite degree sequence, and (Ψ) holds for 𝕱, then **D** ^𝕱^ is called a **Bipartite 1F1S** problem.

Everything discussed in this subsection applies to all 1F1S degree sequence problems in simple graphs. However, we are particularly interested in the bipartite case, therefore we will discuss these observations for the bipartite case only. We fix the underlying vertex set *V* = (*U*, *W*). Then **D** ^𝕱^ is a bipartite 1F1S problem where the center *s* of the forbidden star belongs to *U*.

**Lemma 6.**

(i) *The space of all realizations of* **D** ^𝕱^ *is closed under* 𝕱-*compatible swap operations*.
(ii) *The* 𝕱-*compatible swap operations are circular C*_4_- and *C*_6_-*swaps*.

*Proof*. (i) As we saw already that any bipartite 1F1S can be understood as an 1F1S on simple graphs, therefore considering ℱ = 𝕱 ∪ [*U*]^2^ ∪ [*W*]^2^ and applying Theorem 5 for the problem **d** ^ℱ^ proves (i).

(ii) Let us consider any alternating elementary circuit *C* in the symmetric difference △ of two different realizations. There is a vertex *u* ∈ *C* ∩ *U* which is ≠ *s*. There is at most one forbidden chord in 𝕱 which is adjacent to *u*. If *C* has more than 6 vertices, then *C* has at least 4 vertices in *W* therefore there exists a vertex *w* ∈ *C* ∩ *W*, such that *uw* is a chord and *uw* is not in *C*. Therefore the corresponding *C*-swap is not compatible with 𝕱.

As we already mentioned, Tutte’s *f*-factor theorem can always be utilized to find actual graphical realizations of the bipartite 1F1S problem. However, in this special case we can prove a Havel type result (similar to Lemma 1) and can construct a greedy algorithm to produce such realizations.

Consider the bipartite 1F1S degree sequence problem **D** ^𝕱^. If the forbidden star is not empty, then let *u* ≔ *s*. Otherwise let *u* ∈ *U* be any given vertex and denote *N*(*u*) ⊂ *W* the set of those vertices which form chords together with *u*. (It is clear that if *u* ≠ *s* then |*W*|−1 ≤ |*N*(*u*)|.)

**Observation 7.** *For any y* ∈ *N*(*u*) *there is at most one vertex, denoted by y* ^𝕱^, *such that yy* ^𝕱^ *is a non-chord, so it belongs to* 𝕱. *Furthermore if y, z* ∈ *N*(*u*) *and y* ^𝕱^ = *z* ^𝕱^ *then y* = *z*.

Now a linear order ≺_*u*_ on *N*(*u*) is called **good** if it satisfies the following properties: for *y*, *z* ∈ *N*(*u*) and *y* ≺_*u*_ *z* we have

*d*(*y*) ≥ *d*(*z*) and in case of *d*(*y*) = *d*(*z*) we also have *d*(*y* ^𝕱^) ≥ *d*(*z* ^𝕱^).

It is obvious that there always exists a good order on *N*(*u*). Furthermore whenever *d*(*y*) = *d*(*z*) and *d*(*y* ^𝕱^) = *d*(*z* ^𝕱^), then there are more than one good order.

**Lemma 8.** *Let G be a graphical realization of the 1F1S sequence* **D** ^𝕱^, *let u* ≔ *s if the forbidden star is not empty and take any u* ∈ *U otherwise. Let y, z* ∈ *N*(*u*) *where y* ≺_u_ *z with uz* ∈ *E while uy* ∉ *E. Then there exists an alternating chord-cycle C of length at most 6 in G with y, u, z* ∈ *C. Processing C with* 𝕱-*compatible swap operations, we have* Γ_*G*′_(*u*) = Γ_*G*_(*u*) ∖ {*z*} ∪ {*y*} *in the acquired new realization*.

*Proof*. We have *uz* ∈ *E* but *uy* ∉ *E*. At first assume that there exists a vertex *μ* ∈ *U* ∖ {*u*}, such that *μy* ∈ *E*, and *μz* ∉ *E* but *μ* ≠ *z* ^𝕱^. When such vertex exists then 𝓒 = (*u*, *z*, *μ*, *y*) is a suitable alternating chord-cycle.

When *d*(*y*) > *d*(*z*) then there are two vertices *μ* and *μ*′ ∈ *U* such that *yμ* ∈ *E* and *zμ* ∉ *E*, and *yμ*′ ∈ *E* and *zμ*′ ∉ *E*. Now either *zμ* or *zμ*′ is a chord.

However, if *d*(*y*) = *d*(*z*) then it can happen that *z* ^𝕱^ *y* ∈ *E* and

$$\text{for}\;\text{all}\;x\in U\setminus \{u,y^{F},z^{F}\}\;\;\text{we}\;\text{have}\;\;xy\in E\Leftrightarrow xz\in E.$$ (2)

It is important to observe that in this case *y* ^𝕱^ *z* ∉ *E*, otherwise some *x* would not satisfy Eq (2) (in order to keep *d*(*y*) = *d*(*z*)).

So the only case when we do not find automatically an appropriate circular *C*_4_-swap with *u*, *y* and *z* is when *d*(*y*) = *d*(*z*), *yz* ^𝕱^ is an edge and *zy* ^𝕱^ is a chord but not an edge. In this case, we can find a *μ* ∈ *W* ∖ {*y*, *z*} such that *y* ^𝕱^ *μ* ∈ *E* but *z* ^𝕱^ *μ* ∉ *E* since *d*(*y* ^𝕱^) ≥ *d*(*z* ^𝕱^). Observe that *z* ^𝕱^ *μ* is a chord because *μ* ^𝕱^ ≠ *z* ^𝕱^.

Now 𝓒 = (*y*, *u*, *z*, *y* ^𝕱^, *μ*, *z* ^𝕱^, *y*) is the required alternating chord circle. When *uμ* is a chord, then the circular *C*_6_-swap is not 𝕱-compatible, but we can process the cycle properly (as it was shown in the proof of Lemma 4). When it is a non-chord, then the circular *C*_6_ is an 𝕱-compatible operation.

Lemma 8 provides the following easy Havel type greedy algorithm to decide whether our bipartite 1F1S restricted degree sequence is graphical. (The algorithm is essentially the same as the original Havel procedure.)

**A greedy algorithm** to decide whether a bipartite 1F1S degree sequence is graphical: the degree sequence is **D** while the set of forbidden edges is 𝕱.

(H_1_) Let *u* ≔ *s* if the forbidden star is not empty, otherwise take an arbitrary *u* ∈ *U*. Consider a good order ≺_*u*_ on *N*(*u*). Connect *u* the first *d*(*u*) vertices from (with respect to ≺_*u*_) of *N*(*u*). If *d*(*u*) > |*N*(*u*)| then the algorithm FAILS, our sequence **D** ^𝕱^ is not graphical. Delete *u* from *U*, and update the degree sequence and the set *W* accordingly. Finally delete the edges adjacent to *u* from 𝕱.
(H_2_) Repeat the previous step while *U* is not empty.

**Theorem 9** (Generalized Havel theorem for the bipartite 1F1S ReDeSe problem). *The 1F1S restricted degree sequence* **D** ^𝕱^ *is graphical if and only if the previous greedy algorithm provides a realization*.

*Proof*. The proof is exactly the same as in the original case, described by Havel; if the sequence is graphical, then consider a realization. Fix vertex *u* ∈ *U* as described in Lemma 8. Recursive applications of the lemma provide a realization where *u* is connected to the first *d*(*u*) vertices in *N*(*u*) with respect to ≺_*u*_. The repeated application of the previous reasoning finishes the proof.

## Sampling Degree Sequence Realizations with Sinclair’s Multicommodity Flow Method

There are several available methods to sample uniformly the space of all realizations of a given degree sequence. One of these approaches is a Markov Chain Monte Carlo method, proposed by Kannan, Tetali and Vempala (1999, [3]). They consider a local transformation (the swap operation) on the realizations, which in turn defines an irreducible, reversible and aperiodic finite Markov chain on these realizations; at any given realization they choose two independent edges from some probability distribution and perform the corresponding swap operation if it is feasible. They conjecture that the resulted MCMC is *rapidly mixing*. They were studying the particular case when the degree sequence is regular bipartite using Sinclair’s multicommodity flow method.

Their conjecture was proved for regular graphs by Cooper, Dyer and Greenhill (2007, [4]). (Their result does not apply to bipartite graphs, since their version does not allow forbidden edges.) An analogous theorem was proved by Greenhill on regular directed graphs ([5]). Here she proved at first that for regular directed degree sequences circular *C*_4_-swaps alone make the space of the realizations connected, then she gave a strong upper bound on the mixing time. (However, as we saw it earlier, the space of directed realizations are not always connected when using only *C*_4_-swaps.) Finally, in 2013 Miklós, Erdős and Soukup proved ([6]) that the corresponding Markov process is rapidly mixing on each bipartite **half-regular** degree sequence, superseding the original study of Kannan, Tetali and Vempala ([3]).

In this paper we study the realizations of half-regular bipartite 1F1S restricted degree sequences **D** ^𝕱^. The vertex set is (*U*, *W*) where the center of the forbidden star *s* is ∈ *U* and where all vertex in *U* (except possible *s*) have the same degree. The degrees in *W* are not constrained.

The state space of our **Markov chain** is the graph 𝔾 = (*V*(𝔾), *E*(𝔾)) where *V*(𝔾) consists of all possible realizations of our problem, while the edges represent the possible swap operations: two realizations (which will be indicated by upper case letters like *X* or *Y*) are connected if there is a valid 𝕱-swap operation which transforms one realization into the other one (and the inverse swap transforms the second one into the first one as well). Recall that there are two kinds of 𝕱-compatible swap operations: the circular *C*_4_-swaps and certain *C*_6_-swaps (in the latter case opposite vertex pair in the *C*_6_ must be non-chord), Furthermore, these two kinds of operations make the state space connected (see Theorem 5).

The *transition (probability) matrix* *P* of the Markov chain is defined as follows: let the current realization be *G*. Then

(a) with probability 1/2 we stay in the current state (that is, our Markov chain is **lazy**);
(b) with probability 1/4 we choose uniformly two-two vertices *u*_1_, *u*_2_;*v*_1_, *v*_2_ from classes *U* and *W* respectively and perform the swap if it is possible;
(c) finally with probability 1/4 choose three—three vertices from *U* and *W* and check whether they form three pairs of forbidden chords. If this is the case, then we perform a circular *C*_6_-swap if it is possible.

The swaps moving from *G* to its image *G*′ is unique, therefore the probability of this transformation (the *jumping probability* from *G* to *G*′ ≠ *G*) is:

Prob(G→bG′)≔P(G′|G)=14·1(|U|2)(|W|2), (3)

and

Prob(G→cG′)≔P(G′|G)=14·1(|U|3)(|W|3). (4)

(These probabilities reflect the fact, that *G*′ should be derived from *G* by a regular swap or by a *C*_6_-swap.) The probability of transforming *G* to *G*′ (or vice versa) is time-independent and *symmetric*. Therefore *P* is a symmetric matrix, where the entries in the main diagonal are non-zero, but (probably) distinct values. Our Markov chain is *irreducible* (the state space is connected), and it is clearly aperiodic, since it is lazy. Therefore, as it is well known, the Markov process is reversible with the uniform distribution as the globally stable stationary distribution.

Our main result is the following:

**Theorem 10.** *The Markov process defined above is rapidly mixing on each bipartite half-regular 1F1S restricted degree sequence.*

**Remark 11.** When we apply this setup for directed graphs then the out-degrees are regular (except, perhaps, the out-degree of the vertex *s*), while we have no constrains on the in-degrees. However, it is important to see, that while this result provides a rapidly mixing sampling procedure on regular directed graphs as well, the applied Markov chain is not the same as the one in Greenhill’s model. Hence, this result does not supersede Greenhill’s result.

The proof of Theorem 10 follows closely the proof developed in paper [6]. (More precisely we need to slightly generalize it. The required minor technical issue will be discussed in the Section “Some further technical details of the Sinclair’s method”.) Consider two realizations *X* ∈ 𝔾 and *Y* ∈ 𝔾 of the problem **D** ^𝕱^, and take the symmetric difference Δ = *E*(*X*)Δ*E*(*Y*). As we saw already in the proof of Theorem 5 for each vertex *v* in the bipartite graph (*U*, *W*; Δ) the number of adjacent *X*-edges (= *E*(*X*) ∖ *E*(*Y*)) and the number of the adjacent *Y*-edges are equal. Therefore Δ can be decomposed into alternating circuits and later into alternating cycles. The way the decomposition is executed is described in details in Section 5 of the paper [6]. Here we just summarize the high points:

At first we decompose the symmetric difference Δ into alternating circuits on all possible ways. In each cases we get an ordered sequence *W*_1_, *W*_2_, …, *W*_*κ*_ of circuits. (Usually there are a huge number of different circuit decompositions.) Each circuit is endorsed with a fixed cyclic order.

Now we fix one circuit decomposition. Each circuit *W*_*i*_ from the ordered decomposition determines one unique alternating cycles decomposition: $W_{i}=C_{1}^{i},C_{2}^{i},\ldots ,C_{k_{i}}^{i}.$ (This unique decomposition is one of the most delicate points of the entire proof in [6]. The main problem is that a circuit can be “long”—linear in the number of vertices—therefore, it can happen that it is decomposed into a linear number of cycles. Keeping track of all possible changes along the circuit is necessary, and without clever data handling it may require an unacceptable big data set. Section 5.2 in paper [6] found a way around this problem.)

The ordered circuit decomposition of Δ together with the ordered cycle decompositions of all circuits provide a well defined ordered cycle decomposition *C*_1_, …, *C*_ℓ_ of Δ. This decomposition does not depend on any 𝕱-compatible swap operations (actually no swap operation was performed yet), only on the symmetric difference of realization *X* and *Y*. So this part of the original proof can be used freely in our current reasoning without any modification.

This ordered cycle decomposition singles out ℓ−1 different realizations *H*_1_, …, *H*_ℓ−1_ of **D** ^𝕱^ with the following property: for each *j* = 0, …, ℓ−1 we have *E*(*H*_*j*_)Δ*E*(*H*_*j*+1_) = *C*_*j*+1_ if we apply the notations *H*_0_ = *X* and *H*_ℓ_ = *Y*. This mean that

$$\begin{array}{c} E\left(H_{i}\right)=E\left(X\right)△(\underset{i^{\prime }\leq i}{\cup }E\left(C_{i^{\prime }}\right)). \end{array}$$

What remains is to design a unique canonical path from *X* to *Y* determined by the circuit decompositions which use the realizations *H*_*j*_ as *milestones* along the path. With other words, for each pair *H*_*j*_, *H*_*j*+1_ we have to design the actual swap sequences which turn one milestone into the next one.

So, the canonical path under construction is a sequence *X* = *G*_0_, …, *G*_*i*_, …, *G*_*m*_ = *Y* of realizations, where each *G*_*i*_ can be derived from *G*_*i*−1_ with one feasible circular *C*_4_- or *C*_6_-swap operation, and there exists an increasing subscript subsequence 0 = *n*_0_ < *n*_1_ < *n*_2_ < ⋯ < *n*_ℓ_ = *m* such that we have *G*_*n*_*i*__ = *H*_*i*_.

In paper [6] the following result was proved:

**Theorem 12** (Section 4 in [6]). *If the designed canonical path system satisfies the three (rather complicated) conditions below, then the MCMC process is rapidly mixing. The conditions are:*

(Θ) *For each i* < ℓ *the constructed path* $H_{i}=G_{0}^{\prime },G_{1}^{\prime },\ldots ,G_{m^{\prime }}^{\prime }=H_{i+1}$ *satisfies that* *m*′ ≤ *c*⋅|*C*_*i*+1_| *for a suitable constant c*.
(Ω) ∀*j there exists a K*_*j*_ ∈ *V*(𝔾) *s.t.* $𝔡\left(M_{X}+M_{Y}-M_{G_{j}^{\prime }},M_{K_{j}}\right)\leq \Omega_{2}$, *where M*_*G*_ *is the* **bipartite adjacency matrix** *of G, and* 𝔡 *stands for the Hamming distance of two matrices, finally* Ω_2_ *is a small constant*.
(Ξ) *For each vertex* $G_{j}^{\prime }$ *in the path under construction the following three objects together uniquely determine the realizations X, Y and the path itself*.
  - *The value of the auxiliary matrix* $M_{X}+M_{Y}-M_{G_{j}^{\prime }}$;
  - *the symmetric difference* Δ = *E*(*X*)△*E*(*Y*);
  - *finally a polynomial size parameter set* 𝔹.

The meaning of condition (Ξ) is that these structures can be used to control certain features of the canonical path system: namely their numbers gives a bound on the number of canonical paths between any realization pairs *X*, *Y* which go through any given realization $G_{j}^{\prime }.$ Then condition (Ω) ensures, that the overall number of the used auxiliary matrices is small.

So while we determine our canonical paths among any pair *X*, *Y* we have take care for these three conditions. We will describe the construction itself in the following two Sections.

## The construction of swap sequences between consecutive “milestones”

Now we are going to implement our plan described above. At first we introduce some shorthand. Instead of *H*_*i*−1_ and *H*_*i*_ we will use the names *G* and *G*′. These two graphs have almost the same edge set. More precisely

$$\begin{array}{c} (E\left(G\right)\setminus (C_{i}\cap E\left(X\right)))\cup \left(C_{i}\cap E\left(Y\right)\right)=E\left(G^{\prime }\right)\\ (E\left(G^{\prime }\right)\setminus \left(C_{i}\cap E\left(Y\right)\right))\cup \left(C_{i}\cap E\left(X\right)\right)=E\left(G\right). \end{array}$$

Of course *E*(*G*)Δ*E*(*G*′) = *C*_*i*_ also holds. We refer to the elements of *C*_*i*_ ∩ *E*(*X*) as *X*-edges, while the others are *Y*-edges. We denote the cycle itself by 𝓒, it has 2ℓ edges and its vertices are *u*_1_, *w*_1_, *u*_2_, *w*_2_, …, *u*_ℓ_, *w*_ℓ_. Since 𝓒 has at least four vertices, therefore we may assume that *u*_1_ ≠ *s* (thus *u*_1_ is not the center of the forbidden star). Finally, w.l.o.g. we may assume that the chord *u*_1_ *w*_1_ is a *Y*-edge (and, of course, *w*_ℓ_ *u*_1_ is an *X*-edge).

We are going to construct the realizations $G_{j}^{\prime }$ one by one. We build our canonical path from *G* toward *G*′. At any particular step the last constructed realization is denoted by *Z*. (At the beginning of the process we have *Z* = *G*.) We are looking for the next realization, denoted by *Z*′.

Before we continue the discussion of the canonical path system, we introduce our control mechanism, mentioned in condition (Ω). This *auxiliary* structure originally was introduced by Kannan, Tetali and Vempala in [3]:

For any particular realization *G* from *V*(𝔾) the matrix *M*_*G*_ denotes the *adjacency matrix* of the bipartite realization *G* where the columns and rows are indexed by the vertices of *U* and *W* respectively (Therefore the column sums are the same in each realization, except perhaps at column *s*.) Our indexing method is a bit unusual: the columns are numbered from left to right while the rows are numbered from bottom to the top. (Like in the Cartesian coordinate system.) This matrix is not necessarily symmetric, and elements *M*_*i*,*i*_ can be different from 0.

For example, if we consider the submatrix in *M*_*G*_ spanned by *u*_1_, …, *u*_ℓ_ and *w*_1_, …, *w*_ℓ_ then we have *M*_*G*_(*i*, *i*) = 0 for *i* = 1, …, ℓ, while *M*_*G*_(*i*, *i*−1) = 1 (for *i* = 2, …, ℓ) and *M*_*G*_(1, ℓ) = 1. (So the first value gives the column, the second one gives the row.) The non-chords between vertices in the same vertex class are not considered at all, while non-chords which are forbidden are denoted by ✠. As it is clear from the previous sentence, we will identify each chord or non-chord with the corresponding position in the matrix.

Our auxiliary structure is the matrix

$$\hat{M}\left(X+Y-Z\right)=M_{X}+M_{Y}-M_{Z}.$$

By definition, each entry of a bipartite adjacency matrix is 0 or 1 (or ✠). Therefore only −1, 0, 1, 2 can be the “meaningful” entries of $\hat{M}.$ An entry is −1 if the edge is missing from both *X* and *Y* but it exists in *Z*. It is 2 if the edge is missing from *Z* but exists in both *X* and *Y*. It is 1 if the edge exists in all three graphs (*X*, *Y*, *Z*) or it is there only in one of *X* and *Y* but not in *Z*. Finally it is 0 if the edge is missing from all three graphs, or the edge exists in exactly one of *X* and *Y* and in *Z*. (Therefore if an edge exists in exactly one of *X* and *Y* then the corresponding chord in $\hat{M}$ is always 0 or 1.) One more important, but easy fact is the following:

**Observation 13.** *The row and column sums of* $\hat{M}(X+Y-Z)$ *are the same as row and column sums in M*_*X*_ *(or M*_*Y*_ *or M*_*Z*_).

Next we will determine the swap sequence between *G* and *G*′ through an iterative algorithm. At the first iteration we check, step by step, the positions (*u*_1_, *w*_2_), (*u*_1_, *w*_3_), …, (*u*_1_, *w*_ℓ_) and take the smallest *j* for which (*u*_1_, *w*_*i*_) is an actual edge in *G*. Since (*u*_1_, *w*_ℓ_) is an edge, therefore such *i* always exists. So we may face to the following configuration:

We call the chord *u*_1_ *w*_*i*_ the **start-chord** of the current sub-process and *u*_1_ *w*_1_ is the **end-chord**. We will *sweep* the alternating chords along the cycle from the start-edge *w*_*i*_ *u*_*i*_ (non-edge), *u*_*i*_ *w*_*i*−1_ (an edge) toward the end-edge *w*_1_ *u*_1_ (non-edge)—switching their status in twos and fours. We check positions *u*_1_ *w*_*i*−1_, *u*_1_ *w*_*i*−2_ (all are non-edges) and choose the first chord among them, we call this the **current-chord**. (Since *u*_1_ ≠ *s* therefore we never have to check more than two edges to find the first chord, and we need to check two edges only once, since there is at most one non-chord adjacent to *u*_1_.)

**Case 1**: As we just explained, the typical situation is that the current-chord is the “next” one, so when we start this is typically *u*_1_ *w*_*i*−1_. Assume that this is a chord. Then we can proceed with the swap operation *w*_*i*−1_ *u*_*i*_, *w*_*i*_ *u*_1_ ⇒ *u*_1_ *w*_*i*−1_, *u*_*i*_ *w*_*i*_. We just produced the first “new” realization in our sequence, this is $G_{1}^{\prime }.$ For the next swap operation this will be our new current realization. This operation will be called a **single-step**.

In a realization *Z* we call a chord **bad**, if its current status (being edge or non-edge) is different from its status in *G* (or, what is the same, in *G*′, since they differ only on the chords along the cycle 𝓒). After the previous swap, we have two bad chords in $G_{1}^{\prime },$ namely *u*_1_ *w*_*i*−1_ and *w*_*i*_ *u*_1_.

Consider now the auxiliary matrix $\hat{M}(X+Y-Z)$ (here $Z=G_{1}^{\prime }$). As we saw earlier, for each position outside the chords in 𝓒 the status of that particular position in *Z* is the same as in *X* or *Y* or in both. Accordingly, the corresponding matrix value is 0 or 1. We call a position **bad** in $\hat{M}$ if this value is −1 or 2. (A bad position in $\hat{M}$ always corresponds to a bad chord.) Since in Case 1 we switch the start-chord into a non-edge, it may become 2 in $\hat{M}.$ (In case if in both *X* and *Y* it is an edge. Otherwise it is 0 or 1, so in that case it is not a bad position.) The current-chord turned into an edge. If it is a non-edge in both *X* and *Y* then the value becomes −1, otherwise it does not become a bad position. After this single-step, we have at most two bad positions in the matrix, at most one position with 2-value and at most one with −1-value.

**Fig 1:**
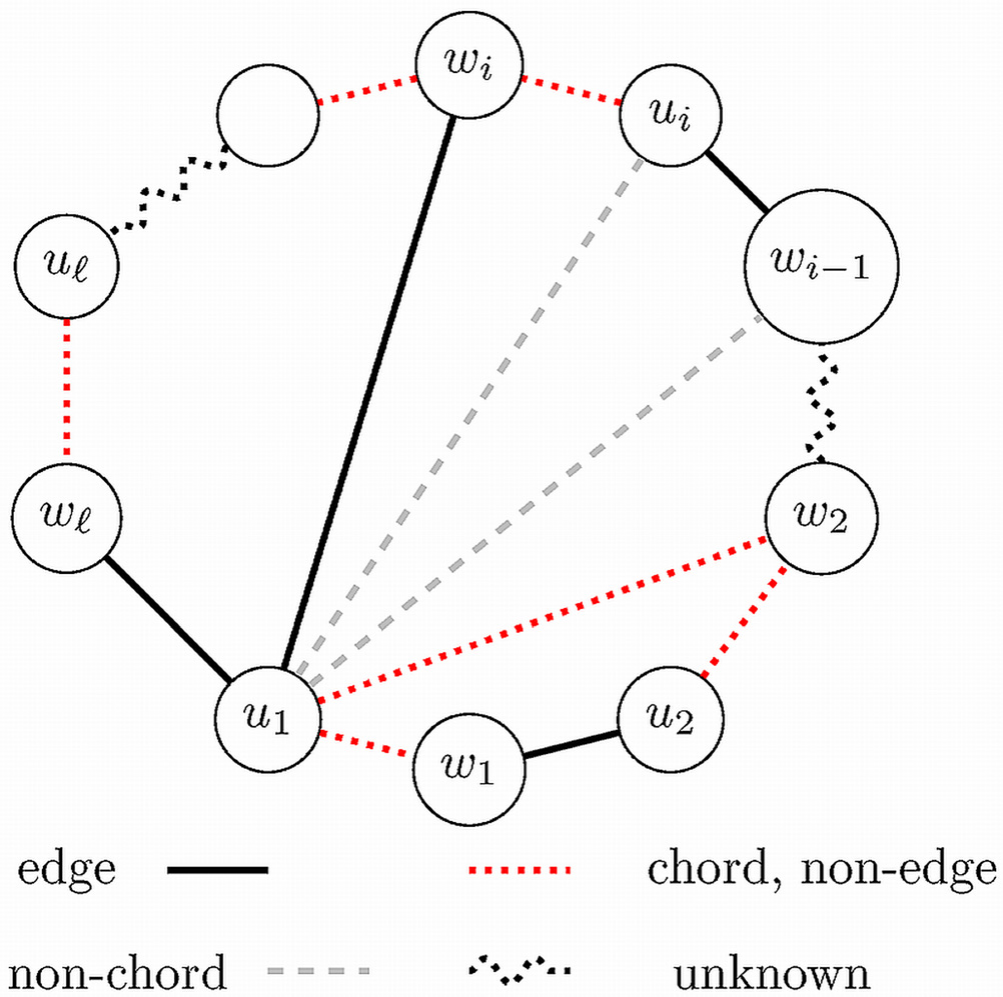
Sweeping a cycle.

**Case 2**: If the previous case does not apply then the pair *u*_1_ *w*_*i*−1_ is a non-chord, therefore we cannot produce the previous swap. Then the non-edge *u*_1_ *w*_*i*−2_ is the current-chord. For sake of simplicity we assume that *i*−2 = 2, this case is represented in Fig 1. Consider now the alternating *C*_6_ cycle: *u*_1_, *w*_2_, *u*_3_, *w*_3_, *u*_4_, *w*_4_. It has a total of three vertex pairs which may be chords. We know already that *u*_1_ *w*_3_ is a non-chord. If none of the three positions is a chord, then this is an 𝕱-compatible circular *C*_6_-swap—and accordingly to the definitions we can swap it in one step. Again, we found the valid swap *w*_2_ *u*_3_, *w*_3_ *u*_4_, *w*_4_ *u*_1_ ⇒ *u*_1_ *w*_2_, *u*_3_ *w*_3_, *u*_4_ *w*_4_. After that we again have 2 bad chords, namely *u*_1_ *w*_2_ and *w*_4_ *u*_1_, and together we have at most two bad positions in the new $\hat{M}(X+Y-Z)$ with at most one 2-value and at most one −1-value.

Finally, if one position, say *w*_2_ *u*_4_, is a chord then we can process this *C*_6_ with two swap operations. If this chord is, say, an actual edge, then we swap *w*_2_ *u*_4_, *w*_4_ *u*_1_ ⇒ *u*_1_ *w*_2_, *u*_4_ *w*_4_. After this we can take care of the *w*_2_, *u*_3_, *w*_3_, *u*_4_ cycle. Along this sequence we never create more, than 3 bad chords: the first swap makes chords *w*_2_ *u*_4_, *w*_4_ *u*_1_ and *u*_1_ *w*_2_ bad ones, and the second one “cures” *w*_2_ *u*_4_ but does not touch *u*_1_ *w*_2_ and *w*_4_ *u*_1_. So along this swap sequence we have 3 bad chords, at the end we have only 2. On the other hand, if the chord *w*_2_ *u*_4_ is not an edge, then we can swap *w*_2_ *u*_3_, *w*_3_ *u*_4_ ⇒ *u*_3_ *w*_3_, *u*_4_ *w*_2_, creating one bad edge, then taking care the four-cycle *u*_1_, *w*_2_, *u*_4_, *w*_4_ we “cure” *w*_2_ *u*_4_ but we switch *u*_1_ *w*_2_ and *w*_4_ *u*_1_ into bad chords. We finished our **double-step** along the cycle.

In a double-step in any moment we have at most three bad chords. When the first swap uses three chords along the cycle then we may have at most one bad chord (with $\hat{M}$-value 0 or −1) and then the next swap switches back the chord into its original status, and makes two new bad chords (with at most one 2-value and one −1-value). When the first swap uses only one chord from the cycle, then it makes three bad chords (changing two chords into non-edge and one into edge), therefore it may make at most two 2-values and one −1-value. After the second swap there will be only two bad chords, with at most one 2-value, and at most one −1-value.

When only the third position corresponds to a chord in our *C*_6_ then after the first swap we may have two −1-values and one 2-value. However, again after the next swap we will have at most one of both types.

**Remark 14.** When two realizations are one swap apart (so they are adjacent in 𝔾) then we say that their auxiliary matrices are at swap-distance one. Since one swap changes four positions of the matrix, therefore the Hamming distance of these matrices is 4.

Finishing our single- or double-step, the previous current-chord becomes the new start-chord. Then we repeat our procedure. There is only one important point to be mentioned: along the step, the start-chord switches back into its original status, therefore it stops being a bad chord. Thus, even if we face a double-step the number of bad chords never will be bigger than three (together with the chord *w*_*i*_ *u*_1_ which is still in the wrong status, so it is bad), and we have always at most two 2-values and at most one −1-value in $\hat{M}(X+Y-Z).$

When *w*_1_ *u*_2_ becomes the current-chord the last step will switch the last start-chord back into its correct status, hence the last current-chord cannot be in bad status. Finally, when the sweep from *w*_*i*_ *u*_1_ to *w*_1_ *u*_1_ is finished we only have one bad chord (with a possible 2-value in $\hat{M}$). This concludes the first iteration of our algorithm.

For the next iteration we seeks a new start-chord between *w*_*i*_ *u*_1_ and *w*_ℓ_ *u*_1_. Chord *w*_*i*_ *u*_1_ becomes the new end-chord. We will repeat our sweeping process for this setup, until all chords are processed. If there was a double-step in the first sweep, then it will not occur again, thus there are never more than three bad chords; at most two 2-values and at most one −1-value.

However, if the double-step occurs sometime later, for example in the second sweep, then we face one of the following two cases: the circular *C*_6_-swap under consideration is either 𝕱-compatible or not. If it is 𝕱-compatible, we perform the circular *C*_6_-swap. This does not change the number of bad chords, except if this swap finishes a current sweep. If, however, the circular *C*_6_-swap is not compatible, then there exists a chord in the chord-cycle which is suitable for a swap. If this chord is a non-edge, then the swap corresponding to it produces one bad chord, and at most one bad position in $\hat{M}.$ If this chord is an edge in the current realization, then after the first swap there are four bad chords, and there may be at most three 2-values and at most one −1 value. After the next swap (which finishes the double step) we annihilate one of the 2-values, and after that swap there are at most two 2-values and at most −1-value along the entire swap sequence. When we finish our second sweep, then chord *w*_*i*_ *u*_1_ will be switched back into its original status, hence it will not be bad anymore.

We apply the same algorithm iteratively. After at most ℓ sweep sequences the entire cycle 𝓒 will be processed. This finishes the construction of the required swap sequence (and the required realization sequence).

Meanwhile we also proved the following important observation:

**Lemma 15.** *Along our procedure each occurring auxiliary matrix* $\hat{M}(X+Y-Z)$ *is at most swap-distance one from a matrix with at most three bad positions: with at most two* 2-*values and with at most one* −1-*value in the same column, which does not coincide with the center of the forbidden star*.

## The Analysis of the Swap Sequences Between “Milestones”

What remains is to show that the defined swap sequences between *H*_*i*_ and *H*_*i*+1_ satisfy conditions (Θ), (Ω) and (Ξ) of Theorem 12. The first one is easy to see, since we can process a cycle of length 2ℓ in ℓ−1 swaps. Therefore the derived constant *c* in (Θ) is actually 1.

Now we introduce the new **switch** operation on 0/1 matrices with forbidden positions: we fix the four corners of a submatrix (none of them is forbidden), and we add 1 to two corners in a diagonal, and add −1 to the corners on the other diagonal. This operation clearly does not change the column and row sums of the matrix. For example if we consider the matrix *M*_*G*_ of a realization of **d** ^𝕱^ and make a valid swap operation, then this is equivalent to a switch in this matrix. The next statement is trivial but very useful:

**Lemma 16.** *If two matrices have* **switch-distance** *1, then their Hamming distance is* 4. *Consequently if the switch-distance is c then the Hamming distance is bounded by* 4*c*.

We prove that property (Ω) holds for auxiliary matrices:

**Theorem 17.** *For any realizations X and Y and for any realization Z on a swap sequence from X to Y there exists a realization K such that*

$$\begin{array}{c} 𝔡(\hat{M}\left(X+Y-Z\right),M_{K})\leq 16. \end{array}$$

Due to Lemmas 15 and 16 it is enough to show that:

**Lemma 18.** *Any matrix* $\hat{M}(X+Y-Z)$ *with constant column sums (this does not necessarily hold for the center of the forbidden star) and with at most three bad positions (where there are at most two* 2-*values and at most one* −1-*value) can be transformed into a valid M*_*K*_ *adjacency matrix with at most three switch operations*.

*Proof*. Consider now a given $\hat{M}$ which is not necessarily a valid adjacency matrix of a realization. We show in figures the submatrix in this matrix that describes the current alternating cycle 𝓒. If it happens that *s* ∈ 𝓒 then we choose a submatrix representation such that the center *s* of the forbidden star is in the first column. (We choose this submatrix as an illustration tool, but we still consider the entire matrix to work with.) We know that this matrix contains at most two 2-values and at most one 1-value. All three positions are adjacent to the center *u*_1_ of the sweeping sequence (see Fig 1), hence they are in the same column.

For simplicity we denote the center of the sweep as well the column with *u*. The forbidden positions are denoted with ✠. Any column (except column 1) may contain at most one of them, and any row may contain at most two of them. Finally, in the figures the character ⋄ stands for a character which we are not interested in. That is, it can be 0 or 1 or ✠.

We distinguish multiple cases, depending on the occurring of values 2 and −1.

**Fig 2:**
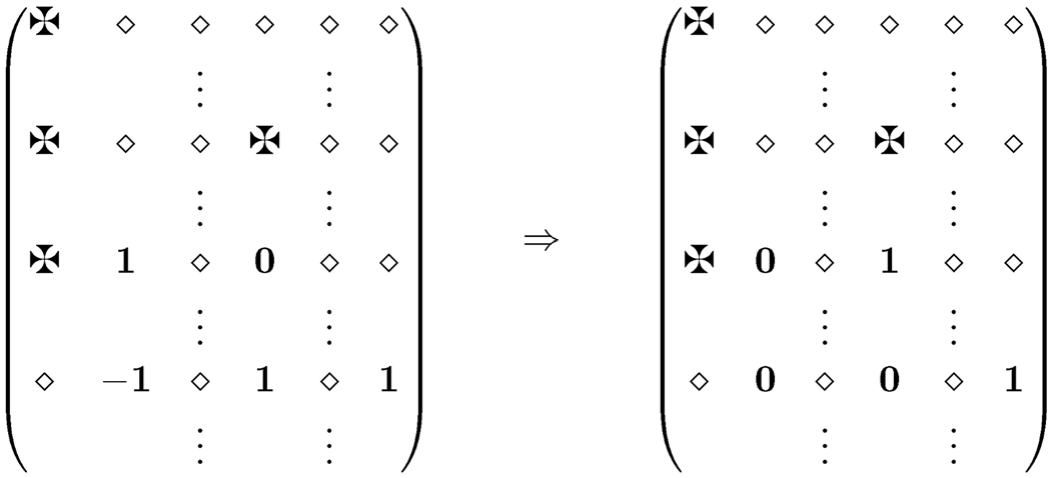
Case 1 ✠ = forbidden ⋄ = 0/1/✠.

**Case 1.** Column *u* has one bad position, which can be −1 or 2, or it has two 2-values. Consider at first the case when $\hat{M}[uw]=-1$. By definition this means that chord *uw* is an edge in *Z* but non-edge in both *X* and *Y*. So vertex *w* ∈ *W* has at least one adjacent edge, therefore the row-sum in its row is at least 1. Therefore there are at least two positions in row *w* with entries 1. They are in column *u*_1_ and *u*_2_. At least one of them, say *u*_1_, differs from *s*. Since the column sums are constant, therefore there exists at least two rows *w*_1_ such that $\hat{M}[uw_{1}]=1$ while $\hat{M}[u_{1}w_{1}]=0$ or ✠. However, there can be at most one forbidden position in *u*_1_, so at least in one of the rows the entry is 0. Using these positions for the corresponding switch it eliminates the bad position without creating a new one. (See Fig 2.)

Before we continue, we prove an important observation:

**Observation** If *w* belongs to the alternating cycle 𝓒 and $\hat{M}[uw]=2$ then row *w* contains at least two 0-values.

Indeed, there are *α* forbidden chords in row *w*. Since *w* is in an alternating cycle, therefore *d*(*w*) ≤ |*U*|−*α*−1. Therefore the sum of row *w* in $\hat{M}(X+Y-Z)\leq |U|-\alpha -1.$ But it contains a 2 and it does not contain -1 therefore there are at least two 0’s in it.

When the single bad value in $\hat{M}$ is 2 then, due to our previous Observation, in its row there are two 0’s. And with them one can repeat the reasoning which we used about the unique −1-value.

Finally, when there are two 2-values which raises a very similar situation. Here we can do the same procedure independently on both rows. In this case, however, we need two switch operations.

**Case 2.** Here we assume that there is one 2-value and one −1-value in column *u*. For example $\hat{M}[uw_{1}]=2$ and $\hat{M}[uw_{2}]=-1$. Again, in row *w*_2_ there are at least two 1-values.

**Fig 3:**
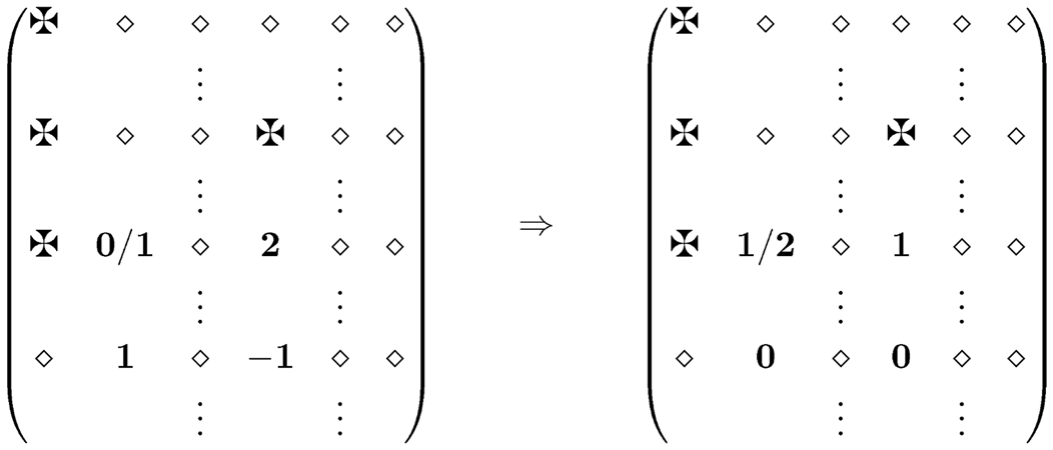
Case 2a ✠ = forbidden ⋄ = 0/1/✠.

*Case 2a* Assume at first that we have *u*_1_ ∈ *U* s.t. $\hat{M}[u_{1}w_{2}]=1$ and $\hat{M}[uw_{1}]\neq ✠$. Then the corresponding switch will produce $\hat{M}[u_{1}w_{1}]=1/2$ while the other three positions are 0 or 1. (See Fig 3.) If now $\hat{M}[u_{1}w_{1}]=2$ then we are back to Case 1, and one more switch eliminates the last bad position as well. So we needed at most two switches.

**Fig 4:**
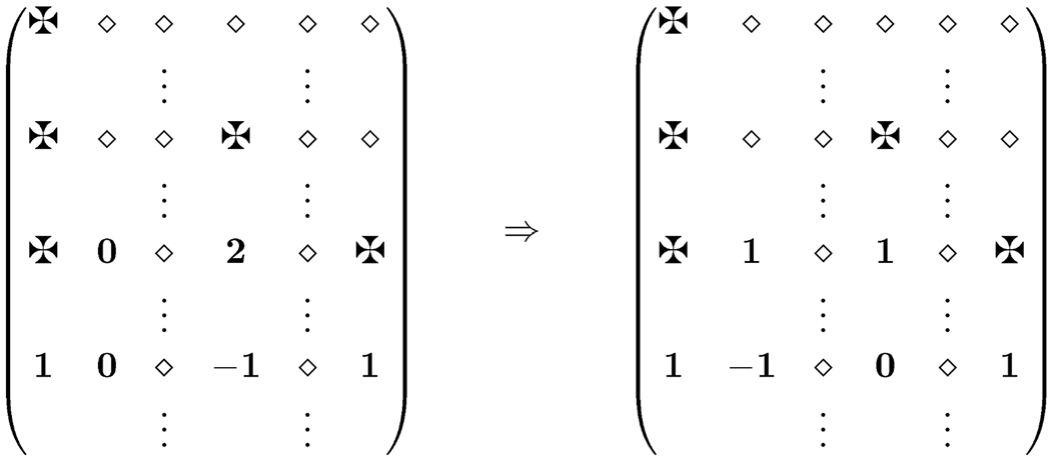
Case 2b ✠ = forbidden ⋄ = 0/1/✠.

*Case 2b* It can happen, that there are only two 1-values in row *w*_2_ and both are facing with forbidden positions in row *w*_1_. Then at least one 0 in row *w*_2_ faces a chord in row *w*_1_. (See Fig 4) The appropriate switch kills 2 bad chords and can make at most one −1-value. At this point we are either finished or back to Case 1.

**Case 3.** Finally suppose that there are three bad positions, two 2-values at positions *uw*_1_ and *uw*_2_ and one −1-value at position *uw*_3_. Now both rows *w*_1_ and *w*_2_ contain at least two 0’s. If any of them face a 1 in row *w*_3_ then an appropriate switch annihilates one 2 and one −1 and does not create new bad position. We are back to Case 1. Altogether we need two switches.

If this is not the case then we consider the following: assume that $\hat{M}[u_{1}w_{1}]=0.$ Since the column sums are the same, and we assumed that $\hat{M}[u_{1}w_{3}]=0$ therefore there exists a row *w*_4_ s.t. $\hat{M}[u_{1}w_{4}]=1$ while $\hat{M}[uw_{4}]=0.$ Then we can switch this 2-value without making a new bad position. After that we are back to Case 2. Altogether this requires at most three switches. The proof of Lemma 18 is finished.

If this is not the case then we consider the following: assume that $\hat{M}[u_{1}w_{1}]=0.$ The column sums are the same, and we assumed that $\hat{M}[u_{1}w_{3}]$ = 0 or ✠. Therefore the difference between column sums in *u* and *u*_1_ is 1 due to rows *w*_1_ and *w*_3_, and the difference increase at least 1 for row *w*_2_, where against a 2-value in column *u* there is either 1 or 0 in column *u*_1_. Therefore there exists at least two further rows, where there is a 1 in column *u*_1_ against a 0 or ✠ in column *u*. Since column *u* can contain at most one ✠, one of the rows must contain a 0. Let it be denoted by *w*_4_. Hence $\hat{M}[u_{1}w_{4}]=1$ while $\hat{M}[uw_{4}]=0.$ Then we can switch this 2-value without making a new bad position. After that we are back to Case 2. Altogether this requires at most three switches. We finished the proof of Lemma 18.

There was no word yet about condition (Ξ) in Theorem 12. We discuss this in the next Section, because the magnitude of parameter 𝔹 heavily depends on. To finish the Theorem 12, let us assume for now that we have the proper upper bound on 𝔹. Then (Ω) in Theorem 12 and therefore the theorem itself is proved as well. Thus, our Markov chain is rapidly mixing as Theorem 10 stated.

## Some further technical details of the Sinclair’s method

In the last two sections we proved the rapidly mixing nature of our proposed MCMC method on the 1F1S restricted degree sequence problems through a special instance of Sinclair’s method, developed in [6]. However, we need slightly generalize this method in order to finish the proof.

Let’s recall that the method takes two realizations, *X* and *Y*, of the same degree sequence. It considers all possible ordered circuit decompositions of the symmetric difference of the edge sets, then it uniquely decomposes each such decomposition into an ordered sequence 𝓒 = *C*_1_, …, *C*_*m*_ of oriented cycles. Based on this latter decomposition the method determines a well defined unique path between *X* and *Y* in the Markov chain 𝔾.

To find this unique path the method first defines a sequence of “milestones”. These are different realizations *X* = *H*_0_, *H*_1_, …, *H*_*m*−1_, *H*_*m*_ = *Y* of the degree sequence where the edge set of any two consecutive realizations *H*_*i*−1_, *H*_*i*_ differ exactly in the edges along the cycle *C*_*i*_. (Until this point no swap operation happened.)

In the next phase, for any particular *i* = 0, …, *m*−1 the method determines a sequence of valid swap operations transforming *H*_*i*−1_ into *H*_*i*_—describing a unique path *Z*_0_, *Z*_1_, …, *Z*_ℓ_ between *H*_*i*−1_ and *H*_*i*_ in the Markov chain 𝔾. This sequence of course depends on the available swap operations. In [6] these are the usual (bipartite) circular *C*_4_-swap operations. In this work these correspond to the restricted swap operations. These operations, while exchanging chords in the realizations along the alternating cycle *C*_*i*_, also use some further chords. Therefore the edge set of any *Z*_*j*_ is not completely contained by *E*(*X*) ∪ *E*(*Y*); there exist a small number of edges in *Z*_*j*_ which are non-edges in *X* and in *Y*, or non-edges in *Z*_*j*_ but are edges in *X* and *Y*. If *Z*_*j*_ is between the milestones *H*_*i*−1_ and *H*_*i*_, then *C*_*k*_ for *k* ≤ *i*−1 alternates in *Z*_*j*_, and *C*_*i*_ alternates with a “small error”: there is a very small number of vertices where the alternation does not hold.

Sinclair’s method requires this number to be small. In the original application this number is actually one (See [6], Section 5, (F)(c).) In the original application this number is actually one. Here, as we saw in Section “Milestones”, this number is three: that many bad chords may occur after any particular ReDeSe. As we saw all these chords are adjacent to the same vertex *u*_1_.

These numbers are used by our method to determine the size of a parameter set 𝔹. This parameter set must have a polynomial size. When we have one bad chord, then it is determined by its end points—there are at most *n* ^2^ possibilities for them. This contributes with an *n* ^2^ multiplicative factor to the size of 𝔹. When we have at most three bad chords, then they can be chosen in at most *n* ^4^ ways: vertex *u*_1_ is fixed (*n* different choices), while the other three end points can be chosen at most *n* ^3^ independent ways. Altogether it contributes with an at most *n* ^4^ multiplicative factor to the size of 𝔹. This remark finishes the proof for the case of 1F1S restricted swap operations.

## 1F1S Restricted Degree Sequence Problem is a Self-Reduced Counting Problem

In Computer Science there are two special complexity classes, FPRAS and FPAUS, which are concerned with the approximability of counting problems. One can find detailed definitions for these complexity classes, for example, in [7]. Here we only give a sketchy description of the points that are important to our case.

Roughly speaking a counting problem is in FPRAS (Fully Polynomial Randomized Approximation Scheme) if the number of solutions can be estimated fast with a randomized algorithm, such that the estimation has a small relative error with high probability.

A counting problem is in FPAUS (Fully Polynomial Almost Uniform Sampler) if the solution can be sampled fast with a randomized algorithm that generates samples following a distribution being very close to the uniform one.

It is easy to see that a counting problem is in FPAUS if there is a rapidly mixing Markov chain for which

- a starting state can be generated in polynomial running time;
- one step in the Markov chain can be conducted in polynomial running time; and
- the relaxation time of the Markov chain grows only polynomially with the size of the problem.

The Markov chain we defined in the 1F1S problem satisfies all these requirements.

Jerrum, Valiant and Vazirani proved that any *self-reducible counting problem* is in FPRAS iff it is in FPAUS [7]. A counting problem is self-reducible if the solutions for any problem instance can be generated recursively such that after each step in the recursion, the remaining task is another problem instance from the same problem, and the number of possible branches at each recursion step is polynomially bounded by the size of the problem instance.

Clearly, a graph with prescribed degree sequence can be built recursively by telling the neighbors of a node at each step, then removing the node in question and reducing the degrees of the selected neighbors. However, this type of recursion does not satisfy all the requirement for being self-reducible since there might be exponentially many possibilities how to select the neighbors of a given vertex.

On the other hand, the degree sequence problem with a forbidden one factor and one star is a self-reducible counting problem. Indeed, consider the center of the (possibly empty) star, *s* ∈ *U*, and the vertex *v* ∈ *V* with the smallest index for which (*s*, *v*) is a chord. Any solution for the current problem instance belongs to one of the following two cases:

- The chord (*s*, *v*) is not present in the solution. In this case, extend the size of the star by adding chord (*s*, *v*) to the forbidden set, and do not change the degrees. This is another problem instance from the 1F1S problem, whose solutions are the continuations of the original problem belonging to this case.
- The chord (*s*, *v*) is present in the solution. In this case, extend the size of the star by adding chord (*s*, *v*) to the forbidden set, and decrease both *d*_*s*_ and *d*_*v*_ by one. The new degree sequence is still a bipartite 1F1S restricted degree sequence which is half-regular in class *U* (except, possibly, at vertex *s*), and the solutions of this new problem extended with the previously decided step provide solutions to the original problem.

Since the 1F1S counting problem is a self reducible counting problem, and we proved that it is in FPAUS therefore it is also in FPRAS: via our sampling process one can solve the approximate counting problem with high probability.

We finish this paper with a short analysis of the connections between our approach and the paper [18] of Jerrum, Sinclair and Vigoda. Their seminal result from 2004 solved the uniform sampling problem of perfect 1-factors of a given graph. As their Corollary 8.1 pointed out this method can be applied for uniform sampling of the set of all possible realizations of a given *f*-factor of a complete graph. It also proves that the problem is in FPAUS therefore in FPRAS as well.

Since the restricted degree sequence problem in general is equivalent to the *f*-factor problem, therefore our 1F1S ReDeSe problem is only a special case of the *f*-factor problem, so the JSV result applies to it. This describes the similarity.

The important differences lay in the swap operations applied in the JSV method and in the Kannan-Tetali-Vempala Markov chain. In the JSV method a special graph 𝔊 is introduced for the sampling via Tutte’s gadgets. Then the swap operations are working on the graph 𝔊 with the unintended result that for a (sometimes very long) sequence of swaps does not change at all the generated *f*-factor. Combining this issue with the known relative slow mixing time of the Jerrum-Sinclair-Vigoda’s Markov chain, the resulted approach in not suitable for any practical application.

Our Markov chain operates in the original graph and each jump provides a new realization of the original degree sequence problem. Therefore our Markov chain is presumably much faster than the JSV chain, furthermore the JSV theorem does not proves the rapidly mixing nature of our Markov chain. Similarly it does not prove that this Markov chain is a self reducible procedure.
